# Genome Sequence of Fungal Species No.11243, Which Produces the Antifungal Antibiotic FR901469

**DOI:** 10.1128/genomeA.00118-15

**Published:** 2015-04-02

**Authors:** Makoto Matsui, Tatsuya Yokoyama, Kaoru Nemoto, Toshitaka Kumagai, Goro Terai, Masanori Arita, Masayuki Machida, Takashi Shibata

**Affiliations:** a Biotechnology Labs, Astellas Pharma Inc., Ibaraki, Japan; b Technology Research Association of Highly Efficient Gene Design (TRAHED), Hokkaido, Japan; c Fermlab, Inc., Tokyo, Japan; d INTEC Inc., Tokyo, Japan; e Computational Biology Research Center, National Institute of Advanced Industrial Science and Technology (AIST), Tokyo, Japan; f National Institute of Genetics, Shizuoka, Japan; g Bioproduction Research Institute, National Institute of Advanced Industrial Science and Technology (AIST), Hokkaido, Japan

## Abstract

Fungal species No.11243 was originally isolated from a decayed leaf sample collected in Kyoto, Japan. It produces FR901469, a 1,3-beta-glucan synthase inhibitor. The genome sequence of No.11243 was determined and annotated to obtain useful information for improving productivity of the effective antifungal agent FR901469.

## GENOME ANNOUNCEMENT

Originally isolated from a decayed leaf sample collected at Ayabe, Kyoto Prefecture, Japan, fungal species No.11243 (No.11243 hereafter) produces a potent, water-soluble antifungal lipopeptidolactone, FR901469 (1). This compound is an inhibitor of 1,3-beta-glucan synthase, exhibiting antifungal activity against Candida albicans and Aspergillus fumigatus, both *in vitro* and *in vivo* (2). Its IC50 against 1,3-beta-glucan synthase in C. albicans is 0.05 µg/ml, over ten times more effective than other echinocandin-like lipopeptides such as aculeacin A, WF11899A, and papulacandin B (1). The structure of FR901469 is a macrocyclic lipopeptidolactone, whose backbone is mnemonically denoted as Acyl-Thr-Ala-Tyr-Val-4OHPro-Thr-Thr-3OHPro-threo3OHGln-Gly-Thr-Orn-OH (C71H116N14O23). It is presumably synthesized by a nonribosomal peptide synthetase (NRPS) (3).

The taxonomic identification of No.11243 has remained uncertain. It is asporogenous and forms pale orange colonies restrictedly on different types of medium. Its aerial hyphae aggregate fascicularly by mucilaginous exudes. For breeding or optimizing cultivating conditions to improve productivity of FR901469, the knowledge of this fungal genome is necessary. Here, we conducted the genome analysis of this industrially interesting fungus strain.

The genomic DNA of No.11243 was obtained by the CTAB DNA extraction method. A TruSeq DNA PCR-Free sample prep kit (Illumina, San Diego, CA, USA) was used for the library preparation, and the samples were sequenced by MiSeq (Illumina). A total of 20,168,767 paired-end reads of 75-bp length were sequenced. The paired-end reads were error-corrected by the Blue algorithm (4), and assembled by SPAdes Genome Assembler version 3.5.0 (5) into 25 contigs. The contigs were filtered by checking the sequence quality and were subsequently gap-filled by SSPACE software for scaffolding (6). The final genome draft consisted of 20 scaffolds, which were subjected to our custom genome annotation pipeline optimized for fungal genomes.

The total length of the No.11243 assembly was 21,736,922 bp with a GC content of 53.9% and 9,694 predicted protein-coding genes. No rRNA gene was detected by RNAmmer (7), and 37 tRNA genes were identified by tRNAscan-SE (8). We found 4 NRPS genes and 4 polyketide synthase (PKS) genes by using antiSMASH version 2.0 (9), and one of them was predicted to be a FR901469-synthesizing NRPS. The NRPS belonged to a cluster of 10 genes; 8 genes were enzymes participating in FR901469 synthesis, and the remaining ones were a transcription factor and a transporter, respectively.

### Nucleotide sequence accession numbers.

This whole-genome shotgun project has been deposited at DDBJ/EMBL/GenBank under the accession numbers DF938580 through DF938599. The versions described in this paper are the first versions.
